# Dose reduction to organs at risk with deep-inspiration breath-hold during right breast radiotherapy: a treatment planning study

**DOI:** 10.1186/s13014-019-1430-x

**Published:** 2019-12-10

**Authors:** Chloe Pandeli, Lloyd M. L. Smyth, Steven David, Andrew W. See

**Affiliations:** 1 Icon Cancer Centre, Level 4, The Epworth Centre, 32 Erin Street, Richmond, Victoria 3121 Australia; 2 Icon Cancer Centre, Mulgrave, Victoria 3170 Australia

**Keywords:** Breast cancer, Deep inspiration breath-hold, Liver, Lung, IMRT, Regional nodal irradiation, Treatment planning

## Abstract

**Background:**

The addition of regional nodal radiation (RNI) to whole breast irradiation for high risk breast cancer improves metastases free survival and new data suggests it contributes additional benefit to overall survival. Deep inspiration breath hold (DIBH) has been shown to reduce cardiac and pulmonary dose in the context of left-sided disease treated with or without RNI, yet few studies have investigated its utility for right-breast cancer. This study investigates the potential advantages of DIBH in local and locoregional radiotherapy for right-sided breast cancer.

**Methods:**

Free-breathing (FB) and DIBH computed tomography datasets were obtained from twenty patients who previously underwent radiotherapy for left-sided breast cancer. Ten patients were retrospectively planned for whole right breast only irradiation and ten patients were planned for irradiation to the whole breast plus ipsilateral supra-clavicular (SC) nodes, with and without irradiation of the ipsilateral internal mammary nodes (IMN). Dose-volume metrics for the clinical target volume, lungs, heart, left anterior descending artery, right coronary artery (RCA) and liver were recorded. Differences between FB and DIBH plans were analysed using Wilcoxon signed-rank tests, with *P* < 0.05 considered statistically significant.

**Results:**

DIBH increased the average total lung volume compared to FB in both breast only and breast plus RNI cohorts (*P* = 0.001). For the breast only group, there was no significant improvement in any ipsilateral lung dose-volume metric between FB and DIBH. However, for the breast plus RNI group, there was an improvement in ipsilateral lung mean dose (18.9 ± 3.2 Gy to 15.9 ± 2.3 Gy, *P* = 0.002) and V20Gy (45.3 ± 13.3% to 32.9 ± 9.4%, *P* = 0.002). In addition, DIBH significantly reduced the maximum dose to the RCA for RNI (11.6 ± 7.2 Gy to 5.6 ± 2.9 Gy, *P* = 0.03). Significant reductions in the liver V20Gy and maximum dose were observed in all cohorts during DIBH compared to FB.

**Conclusions:**

DIBH is a promising approach for right-breast radiotherapy with considerable sparing of normal tissue, particularly when the ipsilateral IMNs are also irradiated.

## Background

Adjuvant whole breast irradiation following lumpectomy is the standard of care for women diagnosed with early stage breast cancer. For women with node-positive or high-risk node-negative disease, the irradiation of regional nodes reduces the rate of both locoregional and distant recurrence [1, 2]. However, the addition of regional nodal irradiation (RNI) increases the volume of underlying normal tissue exposed to radiation, increasing the risk of toxicity.

Deep inspiration breath-hold (DIBH) is a respiratory manoeuvre predominantly used to mitigate the increased risk of late cardiac toxicity for women receiving left-breast radiotherapy [3]. DIBH significantly reduces cardiac dose during whole left-breast irradiation, with or without RNI [4–8]. Lung-sparing benefits are also reported [4, 5, 9]. Importantly, the technique is highly reproducible and stable over the course of treatment [7, 10, 11].

Despite the widespread implementation of DIBH for left-breast radiotherapy, DIBH is rarely used for right-sided treatment and there are few studies exploring the possible dosimetric advantages. Early data suggests cardiac and pulmonary benefits for right-breast cancer patients when RNI is also prescribed [12, 13]. Liver-sparing is also reported [13, 14], however, data remains limited.

The aim of this treatment planning study was to quantify the dose-sparing benefits of DIBH compared to free-breathing (FB) for right-sided breast radiotherapy, with and without irradiation of the regional nodes. Dose-distributions to the lungs, heart, left anterior descending (LAD) artery, right coronary artery (RCA) and liver are reported.

## Methods

### Patient population and study design

Free-breathing and DIBH computed tomography (CT) scans were obtained from twenty patients originally receiving intact left-breast irradiation between January 2016 and November 2017. All patients were retrospectively planned on both DIBH and FB datasets for radiotherapy to the whole right breast (*n* = 10) or whole right breast plus ipsilateral SC nodes, with or without the ipsilateral IMNs (*n* = 10). No extra imaging was required for the study. This retrospective study was approved by the Epworth HealthCare Human Research and Ethics Committee.

Patients were scanned in a supine position with arms raised over head and supported by a personalised vacuum-fixed mould. Patients did not change position between the FB and DIBH scans. Patients received verbal coaching from radiation therapists, as well as visual bio-feedback, during the DIBH scan. The Real-time Position Management™ (RPM) system (Varian Medical Systems, Palo Alto, CA) was used to monitor breathing during both FB and DIBH scans via a reflective marker box placed at the level of the xiphoid process. All patients were able to hold their breath for greater than twenty seconds to accommodate the scan during DIBH. The CT scan length was from the level of the C3 vertebra to 5 cm inferior to the infra-mammary fold with a CT slice thickness of 3 mm. Scans were imported into the Eclipse™ (Varian) planning system for volume delineation and treatment planning.

### Target volume and organ at risk delineation

Target volumes were delineated by one radiation oncologist and independently peer reviewed by a second radiation oncologist prior to treatment planning. The nodal clinical target volumes (CTVs) were defined according to ESTRO consensus guidelines [15]. The breast CTV was defined as the visible breast tissue on CT cropped 5 mm from the skin surface. The planning target volume (PTV) was a 5 mm isotropic expansion of the CTV which was subsequently cropped 5 mm from the skin surface.

Contours for both lungs were generated using an automated segmentation tool and adjusted manually where necessary. The liver and contralateral breast were contoured manually. The heart, LAD and RCA were contoured manually based on the University of Michigan Cardiac Atlas [16]. As patients were scanned without contrast, a 4 mm margin was used for the LAD and RCA contours.

### Treatment planning

FB and DIBH treatment plans were generated for each patient by one radiation therapist to ensure plan quality and consistency across all patients. The prescription dose for all plans was 40 Gy in 15 fractions. Target coverage criteria were in accordance with ICRU recommendations as follows; maximum dose not exceeding 107% of the prescribed dose, coverage of the PTV by the 95% isodose, mean dose to the PTV between 100 and 102% of the prescription dose.

Breast only plans were planned with a three-dimensional conformal technique using a tangential beam arrangement, predominantly using a 6 MV photon beam energy. Low-weighted 10 MV sub-fields were used where necessary to achieve adequate target coverage. Whole breast plus ipsilateral SC (level III and level IV nodes) plans were generated similarly to the breast only plans, however, with the inclusion of an anterior-oblique field for the SC nodal region. The breast plus ipsilateral SC and IMN group (subsequently referred to as breast plus RNI) were inverse planned using six intensity modulated radiation therapy (IMRT) fields with a beam energy of 6 MV. Dose calculation was performed in Eclipse™ (Varian Medical Systems) with the Anisotropic Analytical Algorithm (Version 13.6.26).

The dose-volume objectives for the lungs and heart were aligned to QUANTEC guidelines as follows [17]; lung V20Gy < 30% and mean dose <20Gy, heart V30Gy < 46%, and mean dose < 26 Gy. The maximum dose to the heart was to be kept as low as possible. The mean dose to the contralateral left breast was restricted to less than 2 Gy.

### Plan evaluation and statistical analysis

Dose-volume histograms (DVHs) were generated for all target volumes and organs at risk (OARs) on the FB and DIBH plans. The following dose-volume metrics were recorded: CTV; mean dose and volume receiving 95% of the prescription dose (V95%), total and ipsilateral lung; mean dose, volume receiving 5 Gy (V5Gy) and 20 Gy (V20Gy) and total lung volume, heart; mean dose and maximum dose, LAD; mean dose and maximum dose, RCA; mean dose and maximum dose, and liver; maximum dose and V20Gy.

All analyses were performed using the XLSTAT software package (version 2019.1.1; XLSTAT, New York, NY). Data are presented as mean values ± standard deviation (SD). Wilcoxon signed-rank tests were used to analyse differences in the dose-volume constraints achieved between the FB and DIBH plans. *P* < 0.05, two-tailed, was considered statistically significant.

## Results

**Fig. 1:**
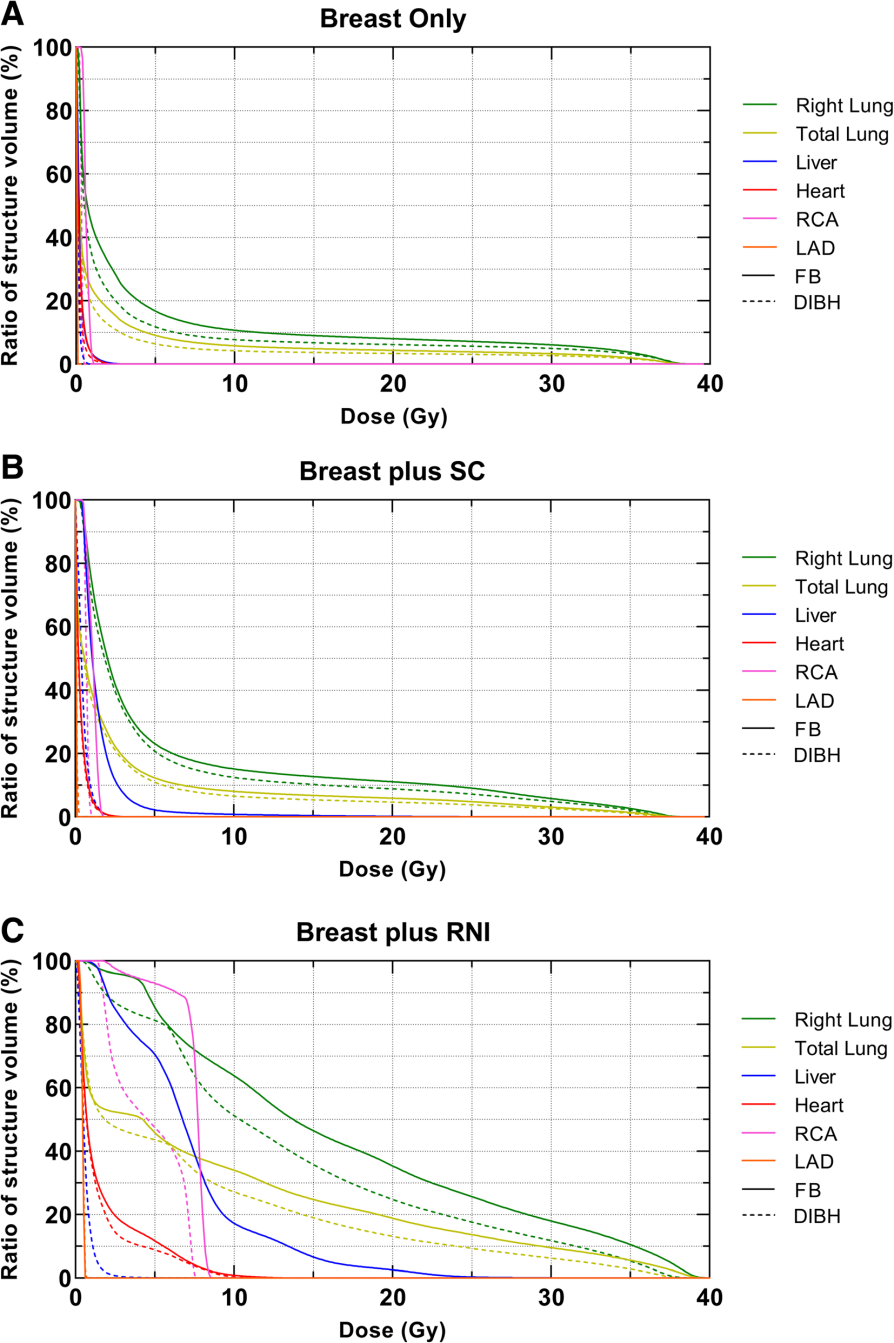
Representative DVHs for OARs when comparing FB and DIBH for breast only (**a**), breast plus SC (**b**) and breast plus RNI (**c**). DVHs taken from one representative patient per group

Dose-volume metrics for target volumes and OARs are summarised in Table 1. Three sets of data were collected; DIBH versus FB for whole right breast only treatment (*n* = 10), right breast plus SC only (*n* = 10) and right breast plus RNI (*n* = 10). A representative DVH comparing FB and DIBH plans for a patient receiving breast only irradiation is shown in Fig. 1a. The DVHs for breast plus SC and breast plus RNI plans, from the same patient, are shown in Fig. 1b and c, respectively. There was no difference in plan quality between DIBH and FB plans for all groups in terms of target coverage (Table 1). All plans, under both breathing conditions, met the target coverage criteria.

**Table 1:** Dose-volume metrics for DIBH and FB treatment plans

Metric	Breast Only			Breast + SC only			Breast + RNI		
	FB	DIBH	*P* value	FB	DIBH	*P* value	FB	DIBH	*P* value
*CTV*									
Mean (Gy)	40.9 ± 0.4	40.9 ± 0.4	0.6	40.9 ± 0.3	40.8 ± 0.3	0.8	40.6 ± 0.2	40.6 ± 0.2	0.5
V95% (%)	99.4 ± 0.7	99.5 ± 0.8	1	99.4 ± 0.4	98.7 ± 1.5	0.3	100.0 ± 0.03	99.9 ± 0.2	1
*Lung (total)*									
Mean (Gy)	2.4 ± 0.5	2.3 ± 0.8	0.3	3.5 ± 0.5	3.1 ± 0.5	0.01	10.8 ± 1.7	8.9 ± 1.2	*0.002*
V20Gy (%)	4.1 ± 0.5	4.0 ± 1.9	0.4	6.7 ± 1.2	5.4 ± 1.2	0.02	25.2 ± 7.6	17.9 ± 5.1	*0.002*
V5Gy (%)	9.6 ± 2.3	8.7 ± 3.2	0.3	13.9 ± 2.3	12.4 ± 1.6	0.02	51.0 ± 3.5	47.4 ± 3.2	*0.004*
Volume (cm^3^)	2613.2 ± 728.1	4467.6 ± 649.0	*0.001*	2714.8 ± 595.0	4452.0 ± 591.5	*0.001*	2714.8 ± 595.0	4452.0 ± 591.5	*0.001*
*Lung (ipsilateral)*									
Mean (Gy)	4.4 ± 1.0	4.3 ± 1.5	0.4	6.3 ± 0.9	5.6 ± 0.9	0.05	18.9 ± 3.2	15.9 ± 2.3	*0.002*
V20Gy (%)	7.6 ± 2.3	7.5 ± 3.6	0.4	12.0 ± 2.2	9.8 ± 2.2	*0.03*	45.3 ± 13.3	32.9 ± 9.4	*0.002*
V5Gy (%)	17.6 ± 4.3	16.4 ± 6.0	0.3	25.1 ± 4.0	22.7 ± 3.1	0.06	91.6 ± 5.6	86.7 ± 7.1	*0.01*
*Liver*									
Max. (Gy)	22.3 ± 15.7	9.2 ± 13.6	*0.006*	32.4 ± 11.0	10.4 ± 10.8	*0.001*	31.3 ± 13.3	15.3 ± 9.8	*0.002*
V20Gy (cc)	9.2 ± 18.9	1.8 ± 5.6	*0.03*	10.8 ± 17.2	0.8 ± 2.6	*0.001*	24.3 ± 38.4	0.8 ± 2.2	*0.02*
*Heart*									
Mean (Gy)	0.2 ± 0.1	0.2 ± 0.1	0.7	0.3 ± 0.1	0.4 ± 0.1	0.2	2.6 ± 0.8	2.1 ± 0.7	0.09
Max. (Gy)	2.4 ± 0.5	2.5 ± 0.4	0.5	1.6 ± 0.5	1.7 ± 0.4	0.9	27.8 ± 7.6	21.5 ± 6.6	*0.02*
*LAD*									
Mean (Gy)	0.03 ± 0.02	0.03 ± 0.02	0.7	0.1 ± 0.04	0.1 ± 0.03	0.2	0.5 ± 0.2	0.6 ± 0.2	0.1
Max (Gy)	0.1 ± 0.1	0.1 ± 0.1	0.4	0.2 ± 0.1	0.2 ± 0.1	0.1	1.2 ± 0.8	1.2 ± 0.7	0.9
*RCA*									
Mean (Gy)	0.7 ± 0.3	0.6 ± 0.3	0.4	0.9 ± 0.2	1.0 ± 0.4	0.5	5.0 ± 2.5	3.5 ± 1.9	0.1
Max (Gy)	1.1 ± 0.4	1.0 ± 0.5	0.5	1.6 ± 0.5	1.7 ± 0.4	0.7	11.6 ± 7.2	5.6 ± 2.9	*0.03*

All data (*n* = 10) are presented as mean ± SD

Abbreviations: *CTV* clinical target volume, *DIBH* deep inspiration breath-hold, *FB* free-breathe, *LAD* left anterior descending artery, *RCA* right coronary artery, *RNI* regional nodal irradiation, *SC* supra-clavicular

### Pulmonary dose

**Fig. 2:**
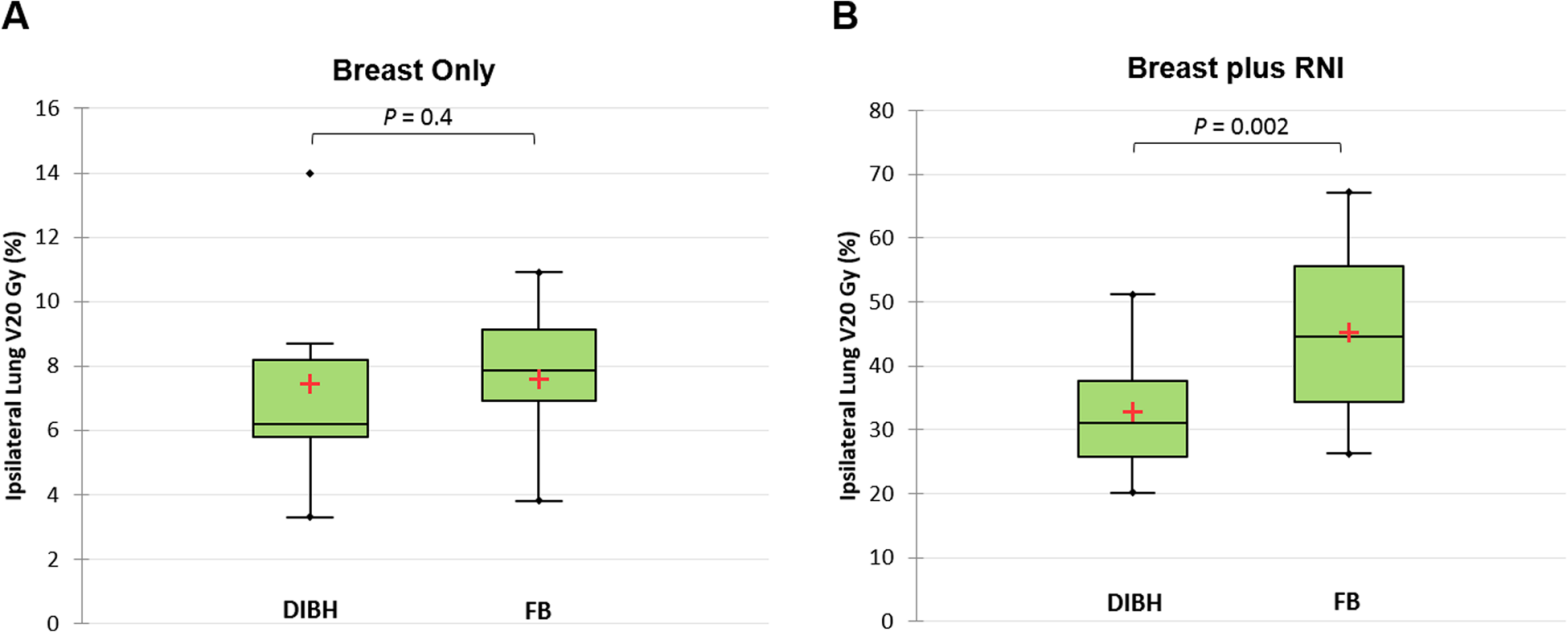
Box-plots of ipsilateral lung V20Gy for DIBH versus FB in right breast only (**a**) and right breast plus RNI (**b**) groups. A significant difference in V20Gy was seen for the breast plus RNI group only. The red cross indicates the group mean and the whiskers extend to 1.5 times the IQR, with *n* = 10 per group

For all three cohorts, there was a similar increase in total lung volume for DIBH compared to FB. The total lung volume for the breast only group increased from 2613.2 ± 728.1 cm^3^ (FB) to 4467.6 ± 649.0 cm^3^ (DIBH) (*P* = 0.001) while for the breast plus RNI and breast plus SC groups, the increase was from 2714.8 ± 595.0 cm^3^ to 4452 ± 591.5 cm^3^ (*P* = 0.001). There was no significant difference in any total or ipsilateral lung dose-volume metric between FB and DIBH in the breast-only group. By contrast, DIBH was associated with significant pulmonary sparing for the breast plus RNI plans. All ten patients in the breast plus RNI group recorded decreases in the mean dose and V20Gy for both total and ipsilateral lung volumes. The ipsilateral lung V20Gy decreased from 45.3 ± 13.3% to 32.9 ± 9.4% for DIBH plans (*P* = 0.002) (Fig. 2), with the largest reduction being from 67.2 to 42.7%. The ipsilateral lung V5Gy decreased from 91.6 ± 5.6% to 86.7 ± 7.1% with DIBH (*P* = 0.01). A significant reduction in the ipsilateral lung V20Gy was also achieved in the breast plus SC group, with 8 out of the 10 patients recording a decrease in this metric.

Two patients in the breast only group had an increase in ipsilateral lung V20Gy with DIBH, however, the constraint of V20Gy < 30% was still achieved. Nine out of ten patients in the breast plus RNI group had an initial ipsilateral lung V20Gy > 30% during FB, with four out of ten meeting this constraint when utilising DIBH.

### Liver dose

**Fig. 3:**
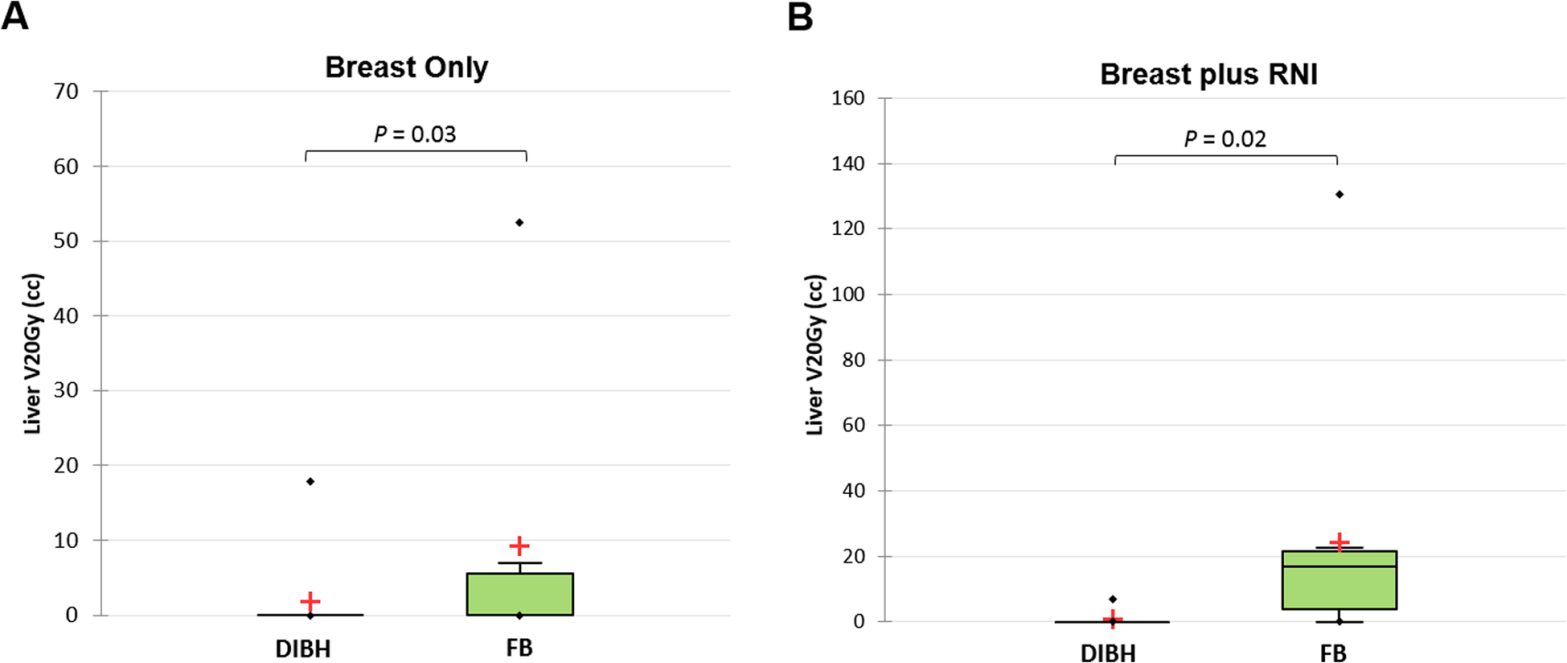
Box-plots of liver V20Gy for DIBH versus FB in right breast only (**a**) and right breast plus RNI (**b**) groups. Statistically significant reductions in the absolute volumes of liver receiving 20Gy were seen with DIBH for both breast only and breast plus RNI groups. The red cross indicates the group mean and the whiskers extend to 1.5 times the IQR, with *n* = 10 per group

**Fig. 4:**
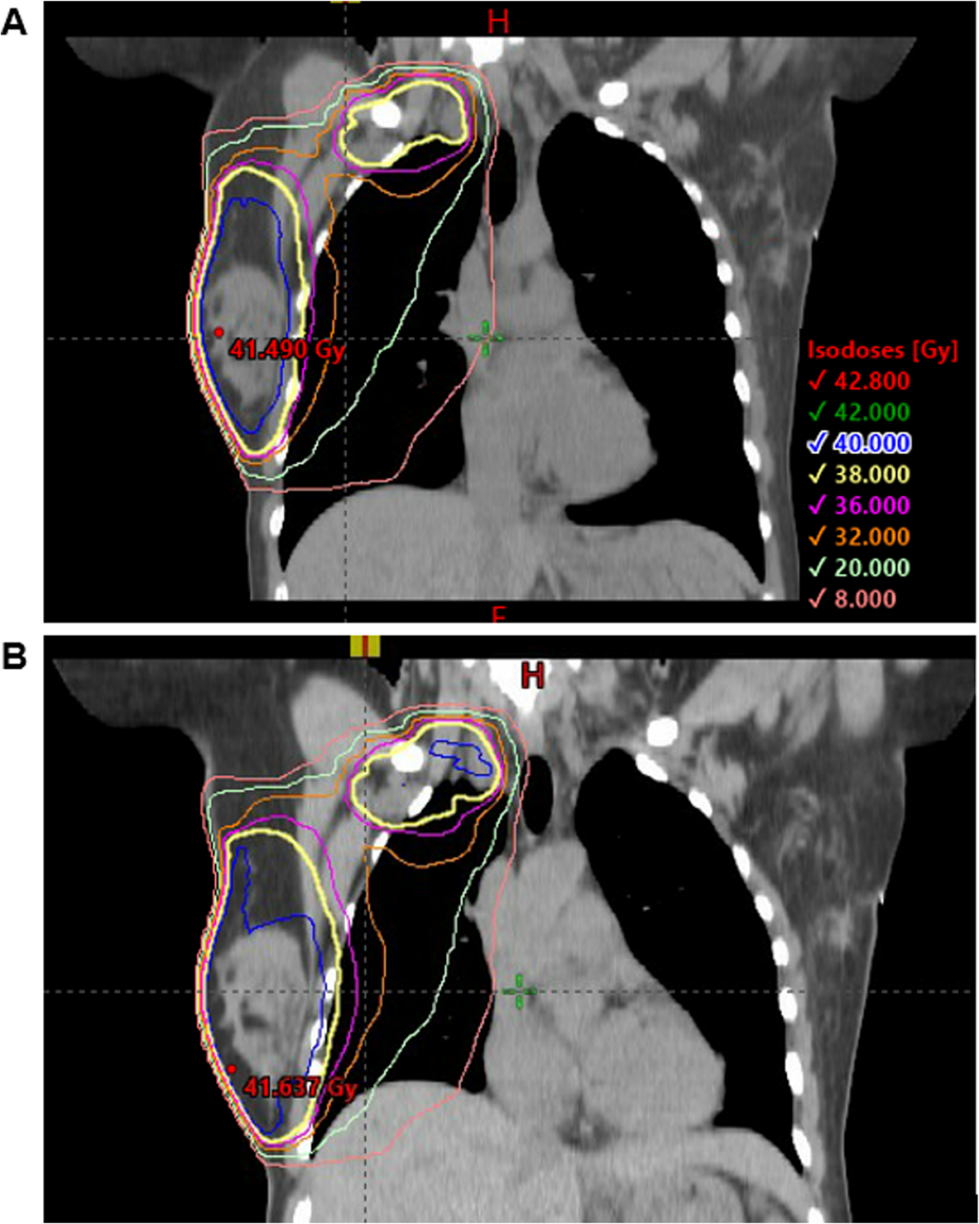
Coronal CT slices demonstrating the superior-inferior liver displacement between DIBH (**a**) and FB (**b**) for a breast plus RNI patient

Statistically significant reductions in the liver V20Gy and maximum dose were observed in both breast-only and breast plus RNI plans during DIBH compared to FB (Fig. 3). The greatest decrease in liver V20Gy was in the breast plus RNI group, with a reduction from 24.3 ± 38.4 cc to 0.8 ± 2.2 cc (*P* = 0.02). The V20Gy was reduced to zero for four out of six patients in the right breast only group and four out of eight patients in the breast plus RNI group. Movement of the liver inferiorly during DIBH compared to FB is shown in Fig. 4 for a pair of breast plus RNI plans from the same patient.

### Cardiac dose

As expected, heart dose was very low for most patients in breast only, breast plus SC and RNI groups. The mean and maximum heart doses for patients in the breast-only group were comparable between FB and DIBH plans. However, a significant decrease in the maximum heart dose was seen for the breast plus RNI plans (27.8 ± 7.6 Gy versus 21.5 ± 6.6 Gy, *P* = 0.02).

In addition, a significant decrease in the maximum RCA dose was observed for the breast plus RNI group, where DIBH decreased the average maximum dose from 11.6 ± 7.2 Gy to 5.6 ± 2.9 Gy (*P* = 0.03). The largest recorded reduction in maximum RCA dose for the RNI group was 21 Gy (FB = 26 Gy, DIBH = 5 Gy). RCA dose was low for the breast only and breast plus SC group in both FB and DIBH.

The dose to the LAD artery was universally low across all groups, with no significant differences between DIBH and FB.

## Discussion

This study supports the use of DIBH for right-sided breast cancer patients, particularly those undergoing concomitant RNI. For patients receiving RNI, there was a 12% and nearly 5% absolute reduction in the volume of ipsilateral lung receiving 20 Gy and 5 Gy, respectively. A significant reduction in both the maximum liver dose and the volume of liver receiving 20 Gy was also observed for right-breast only, breast plus SC and breast plus RNI patients. The maximum dose to the RCA was also significantly reduced for the RNI group.

The rate of radiation-induced pneumonitis (RP) following breast radiotherapy in the modern era is low (1 to 5%) with reductions in lung-function being more common [18, 19]. Nevertheless, increasing pulmonary dose is associated with a higher rate and severity of RP [20]. Unsurprisingly, the rate of RP is higher for breast cancer patients receiving RNI compared to those receiving whole breast radiotherapy only [1, 21, 22]. These observations further support the prudence of utilising DIBH during right-sided breast radiotherapy to reduce pulmonary exposure, which is particularly relevant when the regional nodes are also irradiated. DIBH not only reduces the percentage volume of lung receiving 20 Gy and 5 Gy, but also reduces lung tissue density [23] which could further contribute to a lower normal tissue complication probability [24].

The absolute reduction in ipsilateral lung V20Gy with DIBH in our study (12.4%) was greater than previously reported for right-sided breast cancer patients receiving additional RNI. In this subset of patients, Essers et al. [12] and Conway et al. [13] showed an average absolute reduction in ipsilateral lung V20Gy of 7.5% and of 7.8%, respectively. Our report of a greater pulmonary benefit could be explained by differences in the FB ipsilateral lung V20Gy related to the chosen planning technique. Essers et al. [12] adopted a volumetric modulated arc therapy approach using partial arcs [25] when including the internal mammary and peri-clavicular nodes while Conway et al. [13] used a conformal approach involving wide tangential fields. During left breast irradiation with RNI, the reduction in ipsilateral lung V20Gy is reported to be up to 11.8% [5]. Our finding of a 12.4% reduction in ipsilateral lung V20Gy, with additional RNI, is therefore not unreasonable.

In addition to reducing high-dose pulmonary exposure during RNI, our results show that DIBH also leads to a small yet significant reduction in the proportion of ipsilateral lung receiving 5 Gy. For the complex geometry of the CTV in breast radiotherapy with RNI, intensity modulation optimises dose conformity, homogeneity and target coverage. In our study, conformal coverage of the CTV by the 95% of the prescription dose was achieved. However, the superior geometric target coverage associated with IMRT compared to 3D conformal techniques is offset by increased low-dose exposure due to additional beam angles, monitor units, and inter-leaf radiation leakage. In a meta-analysis of 762,468 breast cancer patients, Grantzau and Overgaard [26] found a significantly increased risk of secondary cancer following radiotherapy for breast cancer. The excess risk of secondary lung cancer in patients receiving radiotherapy compared to those who did not was 39 and 66% at five years and fifteen years following treatment, respectively [26]. Furthermore, the increased use of modern intensity modulated approaches is estimated to nearly double the incidence of secondary malignancies from 1 to 1.75% at ten years following treatment [27]. Therefore, RNI delivered by IMRT - which is the standard at our institution - warrants the utilisation of DIBH to reduce the volume of lung exposed to low-dose irradiation and to potentially offset the additional risk of secondary malignancy.

One of the potentially important findings of our study is the significant reduction in hepatic dose with DIBH for both right-breast only and breast plus RNI groups. Radiation-induced liver damage (RILD) is not commonly associated with breast radiotherapy, but rather with treatments involving the abdomen, lower lobe of the right lung, or distal oesophagus. The dose delivered to the liver during right breast radiotherapy is substantially lower than the mean dose (30–32 Gy) considered to be a significant predictor for a 5% chance of RILD [28]. While the clinical significance of hepatic dose during right-breast radiotherapy remains to be established, it is in the best interests of patients, and in alignment with the ‘as low as reasonably achievable’ (ALARA) principle, to reduce dose to all potential organs at risk as much as possible. Our data, alongside previous studies [13, 14, 29], clearly demonstrates a role for DIBH in reducing hepatic dose which is achieved through increasing the superior-inferior separation between the CTV and the liver as the lungs expand (Fig. 4).

Although cardiac-sparing is most relevant to left-breast radiotherapy, the irradiation of the ipsilateral IMN in right-sided breast radiotherapy can increase the exposure of the heart to radiation. In our study, DIBH led to a statistically significant reduction in maximum heart dose of 6 Gy during breast plus RNI (*P* = 0.02). A modest but non-significant reduction in mean heart dose of 0.5 Gy (*P* = 0.09) was observed, with reductions up to 2 Gy recorded, which could be attributable to unfavourable cardiac anatomy. Darby et al. [3] previously demonstrated that the risk of major coronary events increases linearly with mean heart dose at a rate of 7.4% per Gray, with no threshold dose. Therefore, a subset of women receiving right breast radiotherapy and RNI – particularly those with unfavourable cardiac anatomy or a background of cardiac comorbidities – could benefit from DIBH.

In addition, our study observed a significant reduction in the maximum dose to the RCA with DIBH in the breast plus RNI group. While there is limited data on the effects of radiation on the RCA, a recent study by Altinok et al. [30] suggests that high doses to the proximal RCA could predispose patients to coronary artery disease. Therefore, our study provides further support for implementing DIBH for right breast radiotherapy where RNI is prescribed.

The case for utilising DIBH in right-sided breast plus regional node radiotherapy is supported by its ease of use, reproducibility and cost-effectiveness. Commercial [10, 31], non-commercial [32] and combination [33, 34] DIBH solutions yield systematic and random set-up errors of approximately 2 mm or less [10, 11, 35]. The addition of DIBH to breast radiotherapy can be expected to extend treatment appointments by three to five minutes, with minimal changes to overall patient throughout reported [33]. Nevertheless, the utility of DIBH should be considered against the imaging dose of an extra planning CT scan (one each in FB and DIBH) and the patient’s ability to comply with breath-hold requirements. There are also cases where DIBH may not lead to a material improvement in dose-distribution. In our study, three of twenty patients had an increase in ipsilateral lung V20Gy with DIBH. As such, there is a need to prospectively identify patients who will dosimetrically benefit from DIBH. Anatomic factors such as tumour bed location, lung volume, and the distance of the heart from the chest wall can optimise the selection of patients for DIBH during left-breast radiotherapy, without the need for a CT scan during DIBH [36, 37]. Future studies are required to identify and validate appropriate selection criteria for the use of DIBH during right-sided breast radiotherapy.

There are limitations to our study that should be noted. Firstly, the value of isolated dose-volume metrics as predictors of pulmonary toxicity is contentious given the relative lack of data specific to breast irradiation, the relatively low rate of RP, and the absence of a clear threshold dose for RP [19]. Ho et al. [38] recently found that the lung V20Gy and V5Gy were not predictive for grade three RP for breast cancer patients receiving addition RNI. In contrast, Lind et al. [39] demonstrated through multivariate modelling that the ipsilateral lung V20Gy predicted for radiologic and symptomatic pneumonitis, as well as pulmonary function. In our study, we chose the ipsilateral and total lung V20Gy and mean dose, in alignment with previous studies and QUANTEC recommendations [19]. The V5Gy was analysed to assess the extent of the low dose region, which is particularly relevant for IMRT and the risk of secondary malignancy [26].

Secondly, the results presented in our study are specific to the planning techniques and dose prescriptions used as standard by our department. The magnitude of the dosimetric benefits of DIBH for right-sided breast cancer will vary between intensity-modulated, static, and arc-based approaches, and vary according to institutional target contouring practices and dose-volume objectives. We used the ESTRO contouring guidelines [15] for defining the breast and regional node target volumes. Resultantly, and similarly to Conway et al. [13], the ipsilateral lung V20Gy for both FB and DIBH plans (breast plus RNI) were relatively high, exceeding 30% for six out of ten patients and 40% for two patients, even after DIBH. In our department, a compromise would be made to reduce the ipsilateral lung V20Gy by either reducing chest wall coverage or accepting 80% isodose coverage of the internal mammary node CTV. The benefit of DIBH in right-sided treatment might be diminished in institutions where the FB ipsilateral lung V20Gy is initially lower, which could be the case if less conservative contouring guidelines are followed or clinical compromises on target coverage are made.

Thirdly, only one radiation therapist generated the treatment plans for all patients. While this has the advantage of eliminating the influence of inter-planner variation between treatment plans, there may still be the possibility for planning bias between different patients. Future planning studies could utilise knowledge-based planning solutions such as RapidPlan™ (Varian Medical Systems) to reduce the variability in plans introduced by inter- and intra-planner biases [40].

Finally, this was a retrospective planning study with a relatively small number of patients in each group. Future prospective studies, with larger cohorts, should be designed to more robustly determine the dosimetric benefits of adding DIBH to right-breast radiotherapy, and ultimately, to identify optimal candidates for the technique. Long-term follow-up of toxicity and clinical outcomes will be essential in establishing the true value of DIBH in the context of right-sided breast radiotherapy.

## Conclusion

We have shown that DIBH could lead to substantial sparing of normal-tissue during radiotherapy for right-sided breast cancer patients, particularly those prescribed RNI to reduce the risk of disease recurrence. For patients receiving treatment to the whole right breast plus RNI, the ipsilateral lung V20Gy, V5Gy and mean dose were significantly reduced, along with a significant reduction in the maximum dose to the RCA. Significant reductions in the liver V20Gy and maximum dose were observed for right sided breast cancer patients regardless of whether the ipsilateral IMNs were targeted. Future prospective studies are required to identify which patients will benefit most from DIBH during right-breast radiotherapy and whether improvements to dose-distribution will translate into improved toxicity outcomes.

## Data Availability

The datasets generated and analysed during the current study are available from the corresponding author on reasonable request.

## Abbreviations

CT: Computed tomography
CTV: Clinical target volume
DIBH: Deep inspiration breath hold
FB: Free-breathing
IMN: Internal mammary nodes
IMRT: Intensity modulated radiotherapy
LAD: Left anterior descending artery
OAR: Organ at risk
PTV: Planning target volume
RCA: Right coronary artery
RILD: Radiation-induced liver damage
RNI: Regional nodal irradiation
RP: Radiation-induced pneumonitis
SC: Supraclavicular
SD: Standard deviation
V20Gy: Volume receiving at least 20 Gy
V25Gy: Volume receiving at least 25 Gy
V5Gy: Volume receiving at least 5 Gy
V95%: Volume receiving 95% of the prescription dose
